# Considerations in establishing a post-mortem brain and tissue bank for the study of myalgic encephalomyelitis/chronic fatigue syndrome: a proposed protocol

**DOI:** 10.1186/1756-0500-7-370

**Published:** 2014-06-18

**Authors:** Luis Nacul, Dominic G O’Donovan, Eliana M Lacerda, Djordje Gveric, Kirstin Goldring, Alison Hall, Erinna Bowman, Derek Pheby

**Affiliations:** 1 London School of Hygiene & Tropical Medicine, ITD/CRD/International Centre for Evidence in Disability, K/490, Keppel Street, WC1E 7HT London, UK; 2 Department of Histopathology, Box 235 Level 5 John Bonnett Clincal Laboratories, Addenbrooke’s Hospital, Hills Road, CB1 0QQ Cambridge, UK; 3 The UK Multiple Sclerosis and Parkinson’s Disease Tissue Banks, Hammersmith Campus, Imperial College, 160 Du Cane Road, W12 0NN London, UK; 4 UCL-RFH BioBank, Royal Free Hospital, 1st Floor, Rowland Hill Street, NW3 2PF Hampstead, UK; 5 PHG Foundation, 2 Wort’s Causeway, Cambridge CB1 8RN, UK; 6 Faculty of Health and Society, Buckinghamshire New University, Uxbridge Campus, 106, Oxford Road, Uxbridge, Middlesex UB8 1NA, USA

**Keywords:** Chronic fatigue syndrome, ME/CFS, Ethical issues, Participatory research, Tissue banks, Tissue donors

## Abstract

**Background:**

Our aim, having previously investigated through a qualitative study involving extensive discussions with experts and patients the issues involved in establishing and maintaining a disease specific brain and tissue bank for myalgic encephalomyelitis/chronic fatigue syndrome (ME/CFS), was to develop a protocol for a UK ME/CFS repository of high quality human tissue from well characterised subjects with ME/CFS and controls suitable for a broad range of research applications. This would involve a specific donor program coupled with rapid tissue collection and processing, supplemented by comprehensive prospectively collected clinical, laboratory and self-assessment data from cases and controls.

**Findings:**

We reviewed the operations of existing tissue banks from published literature and from their internal protocols and standard operating procedures (SOPs). On this basis, we developed the protocol presented here, which was designed to meet high technical and ethical standards and legal requirements and was based on recommendations of the MRC UK Brain Banks Network. The facility would be most efficient and cost-effective if incorporated into an existing tissue bank. Tissue collection would be rapid and follow robust protocols to ensure preservation sufficient for a wide range of research uses. A central tissue bank would have resources both for wide-scale donor recruitment and rapid response to donor death for prompt harvesting and processing of tissue.

**Conclusion:**

An ME/CFS brain and tissue bank could be established using this protocol. Success would depend on careful consideration of logistic, technical, legal and ethical issues, continuous consultation with patients and the donor population, and a sustainable model of funding ideally involving research councils, health services, and patient charities. This initiative could revolutionise the understanding of this still poorly-understood disease and enhance development of diagnostic biomarkers and treatments.

## Findings

### Introduction

Myalgic encephalomyelitis (or encephalopathy)/chronic fatigue syndrome (ME/CFS) is classified by the World Health Organization (WHO) as a neurological disorder (ICD10, G.93.3) [1]. Its aetiology and pathogenesis have not been elucidated, although increasing evidence points to nervous system abnormalities [2-11] and immunological dysregulation [12-17]. The diagnosis is clinical and based on a history of unexplained persistent or recurrent incapacitating fatigue for over six months accompanied by variable symptoms, leading to substantial reductions in previous levels of occupational, educational, social and personal activities [18,19], often to moderate or severe disability. There are currently no widely accepted confirmatory diagnostic tests or specific treatments.

Post-mortem examinations have greatly helped to clarify the aetiology and pathogenesis of a wide range of medical disorders, e.g. Creutzfeldt-Jakob disease (CJD) and Parkinson’s disease, opening possibilities for better diagnosis and in many cases treatment. The poor understanding and lack of consensus on the causes and pathophysiological mechanisms involved in ME/CFS and the lack of specific animal models suggest its suitability for study through pathology. However, and rather surprisingly, only a very small number of (ad hoc) pathological studies have been conducted in people who have died with ME/CFS. A few but not all autopsies conducted have shown nervous system abnormalities, including inflammation in the dorsal root ganglia [2] and evidence of viral encephalitis [20,21]. These do not, however, constitute sufficient evidence to confirm the role of the central or peripheral nervous system in its aetiology or pathogenesis.

Calls for brain banks to study ME/CFS have been made in the US [22] and Australia [23] as early as 1996 and 1999. The CFS Peer Reviews for the Centers for Disease Control (CDC) in the US recommended the establishment of a CFS patient brain bank for “neuropathological analysis for tissues not available by other mechanisms” [24]. Nevertheless no action from the CDC or others has yet resulted from that recommendation, and no initiatives have been reported towards systematically addressing the use of pathology for the study of ME/CFS. The only related initiative has come from the Sun Health Research Institute in Sun City, Arizona, US [25], which reported the creation in 1997 of the first and still incipient tissue bank for the study of fibromyalgia, a condition closely related to ME/CFS. A study carried out by two of the authors (LN, EL) confirmed that most patients with ME/CFS favour the establishment of a post-mortem tissue bank [26].

We hypothesise that the better understanding of the aetiology and pathology of ME/CFS following systematic tissue collection and examination would lead to improved diagnosis and disease recognition. Moreover, it would allow the development of new treatment options and interventions specifically targeting possible causes of disease and thus potentially resulting in clinical improvement of patients. This paper discusses considerations in setting up and maintaining a brain and tissue bank for the study of ME/CFS and outlines a protocol for accomplishing this.

The aim of the study is to review the fundamental requirements for and to develop a protocol for establishing an internationally unique facility of high quality human tissue from well characterised subjects with ME/CFS and controls, suitable for a broad range of research applications. This will be achieved through a specific donor program coupled with a system for rapid tissue collection and processing, supplemented by comprehensive prospectively collected clinical, laboratory and self-assessment data from cases and controls.

Procedures will meet high technical and ethical standards and legal requirements in relation to recruitment and follow-up of donors and the collection, handling, preservation and disposal of tissue. The protocol and procedures adopted will be based on the recommendations of the MRC UK brain banks network [27], of which the ME/CFS Tissue Bank will become a member. We will develop close collaboration with other tissue banks to facilitate the supply of control CNS tissue for research, subject to appropriate consents being available.

Specific objectives include:

• To establish a cohort and donor scheme of well characterized cases of ME/CFS and controls for eventual retrieval of nervous system and other tissue post-mortem;

• To establish a post-mortem brain and tissue bank comprising samples from well characterized cases of ME/CFS and controls;

• To enable high quality pathological research in ME/CFS and identify biomarkers; and

• To disseminate the resource to the international research community and other potential users.

The study, which was approved by the London School of Hygiene and Tropical Medicine (LSHTM) Ethics Committee, did not involve any human subjects, so no consent was required or obtained.

### Materials and methods

We developed a protocol for a UK-based repository of tissues from people with ME/CFS. This resulted from extensive discussions with a range of experts and patients and the results of a qualitative study^a^ reported in detail elsewhere [26], which aimed at determining the acceptability and feasibility of establishing a tissue bank for the study of ME/CFS. These were complemented by a review of the literature on tissue bank operation and of internal protocols and standard operating procedures (SOPs) from established tissue banks. This qualitative study was approved by the London School of Hygiene and Tropical Medicine (LSHTM) Ethics Committee and was performed in accordance with the ethical standards laid down in the Declaration of Helsinki 2008. All participants gave prior informed consent to the use of their inputs in the preparation of reports.

### Results

We were able to establish the desirability and feasibility of establishing a tissue bank for the study of ME/CFS and to develop a protocol for its establishment. Our conclusions were that this would best be met by situating the ME/CFS Tissue Bank within an operational tissue bank, thus benefiting from existing infrastructure and experience whilst optimizing the use of resources. This protocol is generic and could be adopted by any appropriate facility within the UK or, with relevant adaptions, internationally. The Addenbrooke’s Hospital Brain and Tissue Bank in Cambridge is very well placed for this purpose due to its long-term experience in tissue banking and previous involvement in ME/CFS research, and is intended as the initial site for the ME/CFS Tissue Bank. In addition, the involvement of patients and their representatives and particularly charities working with people with ME/CFS (PWME) would provide the necessary framework to ensure that the research is truly participatory and ongoing recruitment is safeguarding the long term sustainability of the Bank.

The protocol we developed as a result of this study is detailed below:

### Overview of procedures

#### Sources and procedures for recruitment of tissue donors

Potential donors will be able to access information about the Tissue Bank and targeted-donor scheme primarily from the project webpage and from GPs and other health professionals, including at selected NHS ME/CFS clinics, particularly those situated within the catchment area of the Tissue Bank, i.e. in the East of England. Depending on availability of funding, the intention would be to expand the catchment area to include other regions of the country, with the potential to include other services as receptors of tissues. Other sources of participants may include disease specific charities and support groups, related newsletters and magazines, scientific and group meetings with patients and from GPs and other health professionals, however, these avenues of publicity and any expansion from the geographical area of recruitment, could only be executed in line with the capacity of the team to absorb the potentially high levels of demand for donations. Information packs will be sent to potential donors in response to requests for information. These will include an information booklet, the donor consent form, a form for close relatives of the donor to complete indicating their agreement with the proposed donation, a health questionnaire, our latest newsletter, and a self addressed and stamped or freepost envelope. Potential donors, including cases and controls, will have the opportunity to phone Tissue Bank staff to clarify any doubts before deciding to enrol. In addition, they will have the opportunity to discuss any issues directly with the Tissue Bank of staff collecting their blood samples. Those deciding to register as donors will be issued a donor card containing the contact details of the Tissue Bank and will be asked to inform their close relatives and GPs of their donor status.

#### Inclusion and exclusion criteria for donors

Donors will include individuals over 18 years old with a confirmed diagnosis of ME/CFS for at least two years using the Fukuda (‘CDC-94’) and Canadian Consensus Criteria, which are standard, widely-accepted case definitions [18,19], as ascertained by a health professional with knowledge of ME/CFS, e.g. a specialist working in an ME/CFS clinic or a GP with a special interest in ME/CFS. We will ask patients to confirm they have been diagnosed by a medical professional with expertise in ME/CFS and to include a letter from their GP or another health professional confirming their diagnosis has been formally made. Controls will include individuals of the same age group and without present or past history of chronic fatigue or other neurological, psychiatric, immune and inflammatory diseases or major morbidity, such as cancer. These will be sought from friends, relatives of cases and other volunteers. Since the control donors will be recruited as a direct result of their relationship with affected individuals, recruitment of control donors would be necessarily targeted at those who have knowledge of (and possibly indirect experience of) the condition. We will ensure that consent by control subjects is given freely, that they have sufficient time for reflection, and that they can access more information about what is involved in the Tissue Bank independently of cases in simple and straightforward ways, using the same channels of information and response to queries as cases.

Donors will also be asked to give consent as ‘control subjects’ for studies conducted for other diseases, in connection with other tissue banks in the network. Individuals with confirmed previous ME/CFS, but who are asymptomatic and no longer fulfil diagnostic criteria, will be accepted as donors and form a separate group (controls with previous ME/CFS). The same procedure will apply to the minority of those who had ME/CFS at recruitment, but who no longer have the condition at the time of death. We will encourage recruitment of family members with ME/CFS as cases.

### Procedures for recruitment and follow-up of donors

#### Recruitment procedures

A self-completed recruitment form will establish detailed information on diagnosis, clinical symptoms, potential exposures and socio-demographic variables. Donors will be invited to donate up to 100 ml of blood for freezing and long-term storage^b^, which may be used for specific research projects and as a further source of information for the characterisation of cases post-mortem.

#### Follow–up of donors

Donors will be followed up at least every two years through postal or electronic questionnaires. Information from the questionnaires will be complemented by clinical data, e.g. those provided to the ME/CFS Disease Register and UK ME/CFS Biobank, clinical notes from assessments by GPs, research physicians and specialist doctors, and from laboratory and other tests, where appropriate. This complementary information will usually be obtained post-mortem.

Table 1 illustrates the potential yield of effective tissue donations over 5 years, according to the number of PWME who register as donors (as a percentage of total number of people estimated to have the condition), based on an average UK population mortality. A similar yield for controls of the same age group and sex could be expected within the same periods of time. The actual numbers would depend on resources and interest.

**Table 1:** Expected maximum number of effective tissue donations by time from recruitment in the donor scheme

**Number of donors (% of PWME)**	**1 year**	**2 years**	**5 years**
1,500 (1%)	**3**	**6**	**20**
3,000 (2%)	**6**	**11**	**41**
19,000 (5%)	**15**	**29**	**102**
15,000 (10%)	**30**	**57**	**204**

(PWME = people with ME/CFS).

### Post-mortem procedures

#### Communication of death, inclusion and exclusion criteria

The ME/CFS Tissue Bank will adopt a rapid response system following the death of a registered donor. The system will usually be initiated by a relative or carer telephoning the 24 hr emergency donor line (information found on the donor card), which is linked to a pager system, notifying the Tissue Bank of the death of a registered donor and leaving contact details. In response to the page a member of the Tissue Bank team will immediately call the contact person for further details. An on-call rota system will guarantee that the Tissue Bank will answer every call made to the emergency donor line promptly.

Tissue also will be collected from those who have not previously registered when possible, for example, if family members contact the ME/CFS Tissue Bank team at the time of ‘imminent or actual death’. This will be subject to the requirement of the Human Tissue Act that ‘a decision of [the potential donor] to consent to the activity was in force immediately before he died’^c^[28]. In these cases, donations will be arranged if the documented agreement of relatives and tissue procurement can be organised within the appropriate time frame.

For all donors, tissue donations will not be possible in the following circumstances: (i) if the death has been referred to the coroner and the coroner does not authorise the donation to proceed, or the tissue will not be available in the appropriate time frame, for example due to a prolonged post-mortem interval; and (ii) if it is possible that there is the presence of an infectious disease such as CJD, HIV, MRSA, septicaemia, tuberculosis, or hepatitis B or C.

#### Acquisition and transportation of bodies, tissues and organs to the Tissue Bank

Donors who live in the Tissue Bank area will, in the event of their death, have their whole body transported to the local mortuary for the autopsy and retrieval of relevant tissue samples [29]. On all other occasions tissues will be harvested in a mortuary close to the place of death. Tissues will be either collected as fresh samples from the mortuary by a member of the Tissue Bank team or transported to the tissue bank from the mortuary by authorised couriers following an appropriate period of tissue fixation. We aim to collect our cases within 24 hours of death whenever possible with a 5 day maximum post mortem interval (if refrigerated for histology) to ensure the suitability of tissue for the widest possible range of scientific techniques. Nevertheless we appreciate the difficulties in achieving a very rapid collection of samples, particularly in cases where coroner’s involvement is required, and therefore will consider the inclusion of cases with longer interval periods from death to retrieval of tissues.

#### Tissue preparation and storage

We aim to provide tissue specimens that have been optimally prepared and stored for a variety of techniques as follows:

i. Fresh frozen tissue blocks for mRNA, protein extraction and PCR techniques;

ii. Fresh frozen tissue for cryosectioning for *in situ* hybridisation, immunohistochemistry and cell-based adhesion assays; and

iii. Formaldehyde fixed and paraffin embedded tissue blocks for histology and immunohistochemistry.

For techniques less commonly used, such as ultrastructural studies and cell culture from fresh tissue, arrangements and protocols for tissue provision and collection will be agreed following discussions of requirements with the research investigators concerned.

#### Outline of tissue processing

Immediately upon arrival, tissues will be weighed and the pH of tissue and cerebrospinal fluid (CSF) sample measured. A digital photograph of the lateral, inferior and superior views of the intact brain and lateral views of the spinal cord will be taken at this stage. Any gross pathological changes will be documented. When the brain is processed the whole brain is cut in ‘half’ by a sagittal cut, resulting in half the brain being fixed and half remaining for processing for freezing. In each case at the time of processing a further cut is made through the brainstem to separate the brain stem and cerebellum from the rest of the brain. Then the cerebral hemisphere and the cerebellar hemispheres are sliced and blocked independently. A small piece of tissue (<1 cm^3^) will be taken for total RNA extraction using the guanidinium thiocyanate and CsCl method [30]. This will be used to determine the degree of mRNA preservation for each brain and given an (RIN) number. Following 6 weeks’ fixation, 1 cm thick coronal sections will be sectioned according to international standards [31,32] and digitally photographed before further processing. Blocks from the ‘half’ brain which is frozen will all be photographed and stored. Hence, there is the potential to retrieve samples retained from the whole or one side of the brain. For long-term storage, tissue blocks will be placed in air-tight containers and stored in -80°C freezers with CO_2_ backup and 24 hr monitoring using remote equipment linked to the on-call pager system.

#### Clinical and tissue bank databases

Information gained during the recruitment of donors, the harvesting of brains and the subsequent tissue analysis will be kept securely as three datasets linked in a relational password protected database, which will be accessed only by selected members of the tissue bank team. The first dataset will comprise detailed information of past clinical history (taken from follow-up of donors and complemented by retrospective clinical data obtained from the attending physician’s post-mortem). The GP and specialist notes will be abstracted and added to this dataset. Each donor will be given a number, which will be translated into a brain tissue (donation) number at autopsy.

The second dataset will contain information obtained at autopsy concerning patient details at time of death, macroscopic brain analysis, documentation of slice and block nomenclature. Information obtained from the routine neuropathological screening and further microscopic analysis of tissues will be added to this dataset when it becomes available.

The third dataset will be the image database containing digital images of the gross brain appearance (4 images per brain), images of the whole coronal slices (20–30 images per brain), images of the blocked slices (both fixed and frozen; 20–30 images) and microscopic images of the routine pathological screening.

This database design will allow secure and fast sharing of information and easy transfer of information between datasets. The databases will be fully searchable using adequate query language and updated as required [33].

### Scientific strategy

The post-mortem ME/CFS Tissue Bank team will conduct research with tissues collected from donors and controls, mainly investigating evidence of brain and spinal cord (and its dorsal root ganglia) inflammation and infection. In addition, tissues will be made available to researchers conducting ethically approved studies in the UK and internationally, and following evaluation and approval by a Steering Committee, whose constituents will include professionals with experience in ME/CFS, pathologists, ethicists, patients, and their representatives. Future research will be dictated by advances in scientific knowledge and according to research groups’ interest and expertise. We will develop closer collaboration with other tissue banks in the UK and abroad to facilitate the supply of tissues for both cases and control subjects and to support a wide range of research activities.

### Governance and ethical issues

#### The operation of the tissue bank

The collection and use of human tissue samples for research raises a number of legal and ethical issues. In the past, particular concern has been expressed about the use of post-mortem samples without consent, and in response, legislation has been enacted in the UK to establish a framework for obtaining consent to address this issue [28]. The legislation also establishes systems for licensing tissue banks and for ensuring that the relevant legislation and codes of practice are being adhered to. By using an established tissue bank, the steering committee can be confident that all relevant conditions have been satisfied.

Another means of minimising potential concerns from donors and their families is to promote transparency in the methods and procedures used so as to prevent potential ethical problems; doing so may also optimise recruitment of donors and research outputs. One way this has been achieved to date is by involving patients and other relevant stakeholders in all stages of the process, starting with the planning of the Tissue Bank. This participatory approach will be continued with the implementation and evaluation of the Tissue Bank.

Standard operating procedures will be based on well established procedures and the best available international guidance. Those contracted to collect or process samples on behalf of the tissue bank staff will be required to complete written agreements detailing the procedures for samples to be collected, initial processing and storage, transport of samples to a central storage facility, and the protocol for tissue examination. Strict procedures will be followed in relation to consent, data protection, and other legal and ethical issues, including Human Tissue Authority licensing. Researchers processing tissue from a donor will be required to ensure that a valid consent covering all proposed uses of tissue has been obtained. Researchers will also be required to keep patient identifiable information confidential as a contractual condition.

Electronic sharing of data will be over secure networks, and will, wherever possible, only include coded information on the identity of the donor. Access to these networks will be password protected. All paper records detailing the encryption key will be kept locked on hospital or university premises.

#### Arrangements for follow-up of prospective donors

Although there is a risk that prospective donors might find follow-up intrusive, the arrangements for follow-up will be fully documented in the information booklet provided at the outset. At each point of contact, individuals will be reminded of their continuing rights to withdraw should they so wish. They will also be reminded that they can withdraw without compromising or affecting the medical care that might be available to them. If a potential donor decides to withdraw from the register of prospective donors, any data collected prior to withdrawal, as well as all retrievable biological samples, will be destroyed.

#### Consent form and status of tissue samples

The information and consent forms will make clear to prospective donors that their tissue will be treated as a ‘gift’ to the Tissue Bank and that they will not retain any rights of ownership over the tissue (including any intellectual property rights that may be generated through research on those samples). The consent form will describe in general terms the scope of the possible research uses and the types of tissue and data that might be collected. It will also include information about how samples and data will be encrypted and the measures taken to protect participants’ confidentiality, and on the restricted access of the databases to selected members of the Research and Tissue Bank teams. Where appropriate, it might also refer to samples or data being sent to jurisdictions which have less robust data protection regimes than exist in the UK. Where samples are released to researchers outside the research team, the terms of use will be set out in a licence agreement. This agreement is likely to include that when samples are provided to researchers by the Tissue Bank, it will be on a ‘not for profit’ basis. However, where appropriate, administrative fees may be charged to cover the costs of collecting, processing, handling, and shipping samples, including extra costs incurred in sending samples outside the UK, where applicable. It will also provide that patient confidentiality must be respected.

#### General and ethical oversight

A Steering Group will be formed with members including potential donors with ME/CFS and their representatives, e.g. carers; ME/CFS charity members; other lay-members, such as those from patients and public involvement groups and or others with interest in human research and ethics; experts in pathology, ME/CFS clinical care and research; and lawyers and ethicists with experience in Human Tissue legislation. The group will meet every 4 months to oversee project developments and to deliberate on requests from researchers for tissue provision.

The success of the Tissue Bank will be judged on the number of patients and controls recruited into the donor program, number of donations, quality of post-mortem tissues, number of research projects supplied with tissue, number and quality of publications and communications resulting from such research, and feedback from users and researchers as to the level of service from the Tissue Bank.

### Discussion

The Cambridge Brain Bank, situated at Addenbrooke’s Hospital (Cambridge University Hospitals NHS Foundation) is the longest running Brain Bank in the UK [34]. It was established in 1974 to enable research into nervous system disorders such as dementia (Alzheimer’s, fronto-temporal), motor neurone disease, Huntington’s disease, multiple sclerosis and others.

Since then, various other brain banks have been created and more recently most collections at Addenbrooke’s have been obtained from patients at that hospital and from residents in Cambridgeshire and East Anglia. Its experience in tissue management, status as a centre of reference, and full compliance with Human Tissue Authority regulations make it particularly suitable to host the ME/CFS Tissue Bank, which will benefit from its existing well established infrastructure and resources and also from the infrastructure created for the ME/CFS Disease Register [35]^d^. Membership of the MRC UK will add value to the Tissue Bank and enable it to benefit from and contribute to existing initiatives and proposed developments of the national strategy for the collection of control brain material [27], which is often in short supply.

The still poor understanding of the pathophysiology and aetiology of ME/CFS, added to the difficulties of obtaining tissue samples *in vivo* and the absence of animal models, make it particularly suited for post-mortem pathology studies. This is especially true in light of recent methodological advances in pathology, genomics and proteomics, which enhance the potential of biomedical research. There has been growing evidence pointing to central nervous and autonomic nervous system dysfunctions and disrupted immunity, including impaired functioning of NK-cells and increased levels of pro-inflammatory cytokines. These abnormalities may be triggered by viral infections and other stressors, and possibly by persistent infection. The pathoaetiology of ME/CFS has been reviewed by Shepherd and Chaudhuri [36]. Nevertheless, a very small number of studies on the neuropathology of ME/CFS have been conducted so far. One of the authors (DGOD) at Addenbrooke’s Hospital demonstrated inflammatory changes at the dorsal root ganglia in post-mortem samples of affected patients [37]. The neurosensorial tract changes found are compatible with the central origin pain and fatigue experienced by patients. Further evidence from neurological involvement comes from brain pathology case studies [2,20,21], neuroimaging studies [3,4,7-9,38,39] and the many CNS-type symptoms, such as cognitive dysfunction, hyperacusis, photophobia and headache presented by patients [8,38,40]. However, the abnormalities and mechanisms behind clinical and imaging findings still require elucidation through pathology studies, something that would be enabled by the proposed brain and tissue bank and the generation of a sizable number of donations.

ME/CFS poses unique challenges. It appears to be a heterogeneous condition with no confirmatory diagnostic tests or neuropathological markers. Its study has been hampered by the multitude and lack of specificity of diagnostic criteria in use, which are based mainly on reported symptoms. Good clinical data from donors and the linking of pathological with clinical and laboratory data will enable the search for a pathological biomarker at the same time as the development of better diagnostic criteria and sub-grouping of cases according to clinical and pathological findings.

Our initiative will help address not only the tremendous gaps in knowledge in ME/CFS, but also the general scarcity of CNS ‘control’ tissues, specifically through the sharing of tissues within brain tissue bank networks. The outputs will be enhanced by robust methods, and the use of protocols common to other brain and tissue banks.

For optimum results, we will strive to minimise the post-mortem interval, which for deaths occurring within the geographical area covered by the Tissue Bank will be helped by the relationships developed with local coroners/mortuaries and the possibility of quick transfer of whole bodies for immediate processing by a dedicated team. Moreover, for deaths occurring outside the Tissue Bank coverage area, it will be possible for a member of the research team to travel to the site and rapidly transport material to the Tissue Bank laboratory. This will maximise the potential for a wide range of investigations, including molecular studies and those requiring optimum preservation of the biological and chemical nature of tissue.

This proposal has benefited from extensive discussions with individuals with ME/CFS and charities working in this field. These discussions helped form the donor programme strategy and research protocol and highlighted areas where further debate was required. Participatory research ensures the desires and needs of patients are addressed. It also optimises the acceptability, appropriateness, effectiveness and overall quality of research [26]. ME/CFS charities are already involved in seeking funding for the long term sustainability of the Tissue Bank once support for its implementation from research councils and the NHS is ensured.

### Conclusion

In conclusion, we have established the need for the structured collection and examination of nervous system human tissue of people who have died with ME/CFS. Based on the experience at Addenbrooke’s Hospital and other brain banks, and building on information given by experts and by patients themselves, we have developed a protocol for the first ME/CFS Tissue Bank in the world, including carefully chosen approaches for recruiting and following up donors and for collecting, storing and examining post-mortem tissue samples. This initiative has the potential to revolutionise the understanding of this still poorly recognised disease and greatly help the development of more precise case definitions, diagnostic biomarkers, and treatments.

## Endnotes

^a^The qualitative study included interviews with ‘key informants’ and focus group discussions with people with ME/CFS and the results were reviewed in a workshop with a group of experts, including ME/CFS clinicians, researchers, epidemiologists, pathologists, a lawyer and patient representatives.

^b^These patients will be invited to become part of the linked UK ME/CFS Biobank.

^c^The Human Tissue Act, at s3(6)(c), distinguishes between consent for anatomical examination for which a written consent is required and consent for research for which an oral consent or the consent of a ‘qualifying relative’ is sufficient [28].

^d^This is an ongoing project aimed at the population wide recruitment of people with ME/CFS for clinical and epidemiological studies and at being a source of cases for future research involvement.

## Abbreviations

CDC: Centers for disease control; CJD: Creutzfeldt-Jakob disease; CNS: Central nervous system; CSF: Cerebrospinal fluid; GP: General practitioner; ME/CFS: Myalgic encephalomyelitis / chronic fatigue syndrome; MRC: Medical research council; PM: Post mortem examination; PCR: Polymerase chain reaction; PWME: People with ME/CFS; SOPs: Standard operating procedures.

## Competing interests

The authors declare that they have no competing interests.

## Authors’ contributions

LN, DGOD, EML, DG, KG, AH and DP contributed to the design of the study and the writing of the protocol and first draft of the manuscript. LN, EL, DP and EB contributed to the analysis and interpretation, and all authors read and approved the final version of the paper.

## Authors’ information

Luis Nacul, Eliana Lacerda and Erinna Bowman comprise the CURE-ME team establishing and operating the UK ME/CFS Biobank. Dominic O'Donovan is a consultant neuropathologist from Addenbrooke’s Hospital, Cambridge, with a special interest in ME/CFS. Djordje Gveric is the manager of the Multiple Sclerosis and Parkinson’s Disease tissue banks. Kirstin Goldring is the BioBank and Bio-resources coordinator for the UCL/RFH BioBank. Alison Hall is Programme Lead (Humanities) at the PHG Foundation in Cambridge, with expertise in law and ethics. Derek Pheby was the project coordinator for the National ME/CFS Observatory project, and is the principal investigator and coordinator for the National ME/CFS Disease Register and Visiting Professor of Epidemiology at Buckinghamshire New University.
